# Effect of Impregnation with Natural Shellac Polymer on the Mechanical Properties of Fast-Growing Chinese Fir

**DOI:** 10.3390/polym14183871

**Published:** 2022-09-16

**Authors:** Qinzhi Zeng, Xiya Yu, Nianfeng Wei, Zhiyong Wu, Qisong Liu, Nairong Chen, Weigang Zhao

**Affiliations:** 1 College of Material Engineering, Fujian Agriculture and Forestry University, Fuzhou 350002, China; 2 National Forestry and Grassland Administration Key Laboratory of Plant Fiber Functional Materials, Fuzhou 350002, China; 3 Fujian Provincial Forestry Survey and Design Institute, Fuzhou 350001, China

**Keywords:** fast-growing Chinese fir, shellac, impregnation, response face method, mechanical properties

## Abstract

Fast-growing Chinese fir wood has shortfalls such as loose structure and low strength because it grows faster than natural trees. Resin impregnation is a great way to increase the strength of fast-growing fir. However, the resin used for impregnation is a kind of urea-formaldehyde resin, phenolic formaldehyde resin, melamine formaldehyde resin, and the like, which introduce harmful substances such as formaldehyde or phenolic into the wood. In this paper, Chinese fir wood was impregnated with natural shellac polymer, and the effects of impregnation variables on the mechanical properties of the wood were examined. The increase in strength in compression perpendicular to grain (*SCPG*) of wood samples impregnated with 15% shellac solution achieved a maximum value of 39.01%, but the modulus of rupture (*MOR*) was slightly reduced. The effects of the impregnation pressure, time, and their interaction were investigated by the response surface method (RSM). ANOVA analysis revealed that the impregnation pressure and time and the interaction between the two seemed to have a significant effect on ∆*SCPG.* Based on the response face model, the corresponding optimal parameters obtained are 1.0 MPa and 16.0 min for impregnation pressure and time, respectively. By impregnating fir wood with the above optimal conditions, the *SCPG* increased by 85.78%, whereas the *MOR* decreased by the least amount.

## 1. Introduction

The Chinese fir (*Cunninghamia lanceolata* (*Lamb.*) *Hook*) is a principal coniferous species in the subtropical regions of China, distributed in 16 subtropical provinces, municipalities, and autonomous regions in China [1]. The Chinese fir has had a long plantation history for more than 3000 years in China, according to the historical records [2]. Currently, the national Chinese fir plantation area is 8.95 × 10^6^ ha, with a stock volume of 6.25 × 10^8^ m^3^, providing up to 30% of the logs for China’s timber industry [3,4]. It became China’s most important commercial timber species due to its fast-growing, high-yielding, straight trunk, uniform structure with excellent anti-fungi resistance [5,6]. However, some disadvantages, including loose structure, low strength, and dimensional instability, of fast-growing Chinese fir wood seriously affect the service performance, limiting its utilization in the form of solid timber in engineering constructions, such as furniture and floors [7]. Researchers have attempted to reduce these disadvantages through thermal modification, mechanical compression, resin impregnation, and chemical modification processes [8]. Thermal modification of Chinese fir wood can enhance its performance by reducing water absorption and improving dimensional stability and biological durability, but it reduces its mechanical strength [9]. Although mechanical compression may increase the density, modulus of rupture (*MOR*), and modulus of elasticity (MOE) of the low-density wood, there will always be a certain recovery of compressive deformation after absorption [10,11]. Using a combined process of thermomechanical densification and heat treatment, Li et al. [12] significantly reduced the recovery of Chinese fir wood. Chemical modification methods such as acetylation, esterification, and furfurylation can alter the relationship between wood and moisture, making the wood hydrophobic, thereby improving dimensional stability and reducing decay susceptibility [13]. The resin impregnation method (RIM) has been shown to be one of the most effective solutions for improving the mechanical properties of Chinese fir wood [14,15]. Various synthetic thermosetting resins are commonly used for impregnating wood, such as urea-formaldehyde resin (UF), phenolic formaldehyde resin (PF), melamine formaldehyde resin (MF), etc. [16,17,18]. To improve the physical and mechanical properties of Chinese fir wood, Ma et al. [19] impregnated it with unsaturated polyester resin. However, the RIM either introduces some harmful chemicals into the wood or produces waste that pollutes the environment. As a result, several inorganic chemicals, such as sodium silicate, have been used to impregnate wood [20,21,22], they it will change the pH of the wood and corrode metal furniture hardware. Replacing synthetic resins with natural polymers that are less harmful to wood and the environment is a significant step forward.

Shellac is a natural biocompatible polymer that is commonly used as a protective coating for food and other products [23]. Shellac is a mixture of polyesters and monoesters that are insoluble in water but soluble in ethanol or ether [24]. Because of its excellent film-forming, strong adhesion to the wood surface, and protective properties, shellac is used as a varnish to preserve the surface of wooden products in the fields of wooden furniture restoration and musical instruments [25]. In this article, natural shellac polymer was used instead of synthetic resin to impregnate Chinese fir wood in order to use an environmentally friendly modifier. It was also investigated whether wood could be impregnated with a shellac solution to improve its mechanical properties.

## 2. Materials and Methods

### 2.1. Materials

Twenty-one year old fast-growing Chinese fir logs were purchased from Nanan City, Fujian, China. The fir logs were 5 m long, and the diameter of the small head was about 320~400 mm. The air-dried density of the fir logs was about 0.36 g/cm^3^. The fir logs were sawn by the rift-sawing method into square-section wood strips with a section size of 30 mm × 30 mm in a lumber mill. Bleached shellac with an average molecular weight of about 765 Da was bought from Yunnan Lvchun Shellac Co., Ltd, in Honghe Hani and Yi Autonomous Prefecture of China. Alcohol with a concentration of 95% was purchased from a chemical store in Fuzhou, China, and was produced by Guangzhou Chongwen Chemical Co., Ltd, in Guangzhou, China.

### 2.2. Wood Samples Preparation

**Figure 1:**
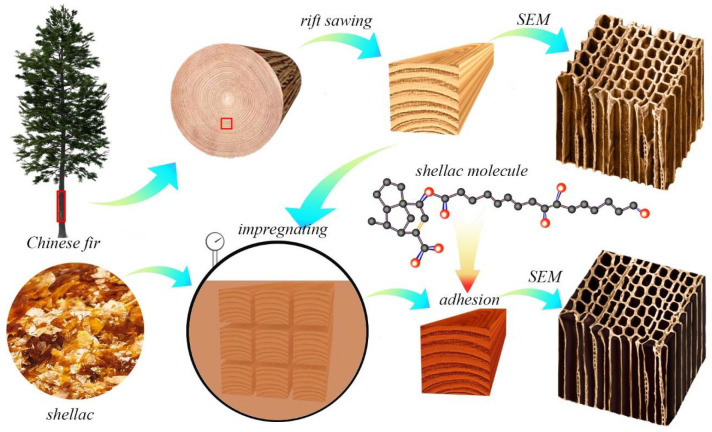
Schematic of the impregnation with shellac polymer.

The wood strips were planed on all four sides and processed into wooden bars with a cross-sectional dimension of 20 mm × 20 mm (the error is 0~2 mm). Next, defects such as cracks, knots, rot, etc., were cut off, and some 300 mm long defect-free wood samples were cut from the wood bars. A pair of wood samples were cut from adjacent locations on the same wood bar: one for the impregnation treatment and the other for the control. The preparation and processing of the specimens is shown in Figure 1.

### 2.3. Microwave Pretreatment

In accordance with the literature, the wood samples were pretreated by microwave [26]. They were immersed in water at 25 °C for 24 h for the impregnation treatment. Subsequently, the wood samples were taken out of the water, and the surface water was wiped away with filter paper. The samples were then pretreated for 100 s in a microwave machine with a microwave power of 4 kW and a frequency of 2.45 GHz.

### 2.4. Impregnation with Shellac Solution of Different Concentrations

Shellac was dissolved in 95% alcohol to prepare 3000 mL shellac solutions with concentrations of 10%, 15%, and 20%.

After drying for 5 h in an oven at 103 ± 2 °C, the wood samples were placed in a pressure kettle. After locking the kettle lid, the pressure in the kettle was reduced to −0.1 MPa by vacuuming, and the shellac solution was sucked into the kettle. Next, the pressure in the kettle was raised to 1.2 MPa using an air compressor. After maintaining the pressure for 30 min, the outlet valve was opened to reduce the pressure and discharge the shellac solution. While the pressure in the kettle was being lowered to atmospheric pressure, the lid of the kettle was opened and the wood samples were taken out. After 24 h of storage at room temperature, both the wood samples and the controls were placed in a constant temperature (20 °C) and humidity (65%) chamber for more than 48 h.

### 2.5. Interaction of Impregnation Pressure and Time

The response surface method (RSM) with two factors and five levels was employed to optimize the experimental conditions of impregnation pressure and time. The concentration of the shellac solution was 15% for RSM analysis, and the specific RSM design is shown in Table 1. The increase rate of the strength in compression perpendicular to grain (Δ*SCPG*), increase rate of the modulus of rupture (Δ*MOR*), and weight gain percent (*WGP*) were used as the response of the RSM. Other parameters and procedures were the same as described above.

### 2.6. Performance Testing

#### 2.6.1. Strength in Compression Perpendicular to Grain

In accordance with ISO 13061-5:2020, the strength in compression perpendicular to grain (*SCPG*) of the wood samples was determined by the radial loading. The loading direction is the radial direction of the wood, and the loading speed is 5 mm/min. The Δ*SCPG* between an impregnated sample and its control sample was calculated by Equation (1):

(1) $$\Delta SCPG_{i}=\frac{SCPG_{Ti}-SCPG_{Ci}}{SCPG_{Ci}}\times 100\%$$

where $\;\Delta SCPG_{i}$ was the *SCPG* increase for the ith pair wood samples; $SCPG_{Ti}$ was the *SCPG* of the ith treatment sample (MPa); and $SCPG_{Ci}$ was the *SCPG* of the control sample adjacent to the ith treatment sample.

#### 2.6.2. Modulus of Rupture

In accordance with ISO 13061-3:2014, the modulus of rupture (*MOR*) of the wood samples was determined by an electromechanical universal testing machine (E44.304, MTS systems Co. Ltd., Eden Prairie, MN, USA). The radius of the support roller and the loading head were 30 mm, and the span was 240 mm. The loading direction was the radial direction of the wood, and the loading speed was 10 mm/min. The Δ*MOR* between an impregnated sample and its control sample was calculated by Equation (2):

(2) $$\Delta MOR_{i}=\frac{MOR_{Ti}-MOR_{Ci}}{MOR_{Ci}}\times 100\%$$

where $\;\Delta MOR_{i}$ was the *MOR* increase for the ith pair wood samples; $MOR_{Ti}$ was the *MOR* of the ith treatment sample (MPa); and $MOR_{Ci}$ was the *MOR* of the control sample adjacent to the ith treatment sample.

#### 2.6.3. Weight Gain Percent

Before impregnation treatment with the shellac solution, the wood samples were dried to absolute dryness in an oven at 103 ± 2 °C, and the mass ($m_{1}$) of each wood sample was weighed by an electronic balance. After impregnation, they were dried to absolute dryness again and their masses ($m_{2}$) were determined. The WGP was computed by Equation (3):

(3) $$WGP=\frac{m_{2}-m_{1}}{m_{1}}\times 100\%$$

#### 2.6.4. Scanning Electron Microscopy (SEM)

After cutting a 5 mm × 5 mm × 2 mm slice from the impregnated wood sample, the distribution of shellac in the wood samples was observed under a scanning electron microscope (SU8010 produced by Hitachi, Tokyo, Japan).

#### 2.6.5. Fourier Transform Infrared (FTIR) Spectroscopy

The samples were pulverized with a ball mill, passed through a 200-mesh sieve, and prepared by KBr tableting method. FTIR spectroscopy was performed on a Fourier transform infrared spectrometer (VERTEX 70, produced by Bruker, Berlin, Germany) to investigate the state of the shellac in the wood. The scanning wavelength range was 4000–500 cm^−1^, the number of scans was 32, and the spectral resolution was 4 cm^−1^.

#### 2.6.6. X-ray Diffraction (XRD)

The crystallinity of the wood samples was characterized by an X-ray diffractometer on a Bruker D8 Advance diffractometer (Cu-K, Bragg-Brentano Geometry, Billerica, MA, USA). The diffraction angle range was 5°~40° (2θ), and the scanning rate was 10°/min. The crystallinity index was calculated from the heights of the amorphous and the total intensity with Segal’s method [27].

#### 2.6.7. X-ray Photoelectron Spectroscopy (XPS)

After drying the sample to absolute dryness with a vacuum dryer, XPS scans were performed by Thermo Scientific (Waltham, MA, USA) K-Alpha to study the shellac molecules present in wood. The vacuum pressure of the analysis chamber was about 5 × 10^−7^ mbar, and the X-ray source was the monochromatic AlKa, 1486.6 eV energy, 12 kV voltage, and 6 mA beam current.

## 3. Results and Discussion

### 3.1. Effect of the Concentration of the Shellac Solution

**Figure 2:**
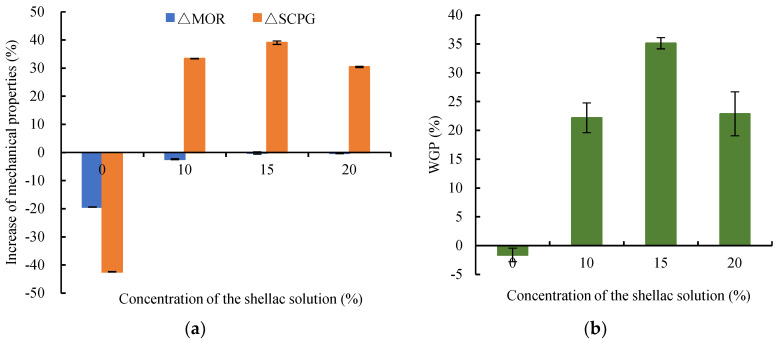
The mechanical strength and weight gain of wood samples impregnated with different concentrations: (**a**) increase in mechanical properties; (**b**) WGP.

As shown in Figure 2a, wood samples impregnated with different shellac concentrations improved both *MOR* and *SCPG*. When impregnated with pure industrial alcohol (0% concentration, as shown in Figure 2), the *MOR* and *SCPG* were reduced by 19.40% and 42.43%, respectively. The WGP of wood samples was reduced by 1.61% due to the extraction of alcohol-soluble aliphatic and terpenoids (Figure 2b). Comparing wood samples impregnated with different shellac concentrations, wood samples impregnated with 15% shellac had the highest Δ*SCPG* of 39.01%. The corresponding WGP as high as 35.12% was achieved. A quite slight but noticeable decrease in *MOR* was observed as the concentration of shellac solution increased in the impregnated wood samples.

**Figure 3:**
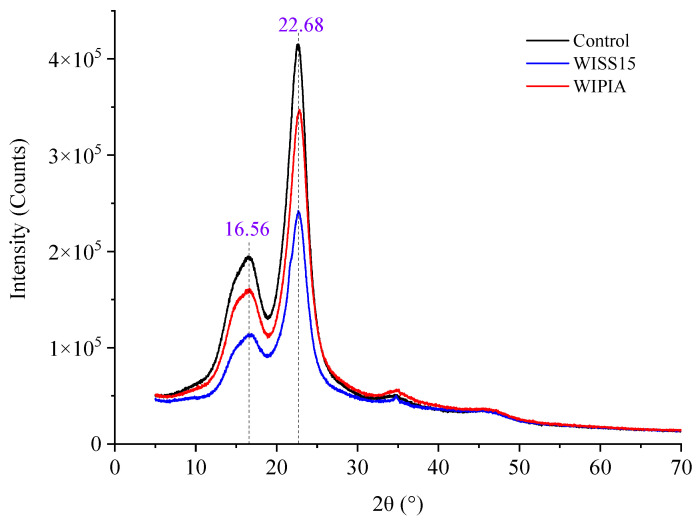
XRD spectra of the wood samples.

Figure 3 shows the XRD spectra of the wood samples impregnated with 15% shellac solution (WISS15), pure industrial alcohol (WIPIA), and a control. There are typical characteristic peaks of cellulose I-type structure at diffraction angles of 16.56° and 22.68° for all the samples [28,29]. WISS15 and WIPIA both exhibit decreased appearance in their diffraction peaks compared with the control sample. The crystallinity of WISS15, WIPIA, and control samples were 53.08%, 52.99%, and 53.49%, respectively. In comparison to control samples, wood samples impregnated with pure industrial alcohol showed a maximum reduction of 0.77% in crystallinity due to the strong permeability of alcohol. The alcohol molecules can enter the interior of the wood under high pressure, even around the crystallization area in the cell wall, and dissolve the alcohol-soluble substances, thereby reducing the crystallinity and mechanical properties. Impregnated with the shellac solution, the solvent alcohol had a similar effect, but the shellac could fill the cell lumen or adhere to the cell wall to improve its resistance to compression. As a result, the Δ*SCPG* of wood samples impregnated with 15% shellac solution increased by 39.01%.

**Figure 4:**
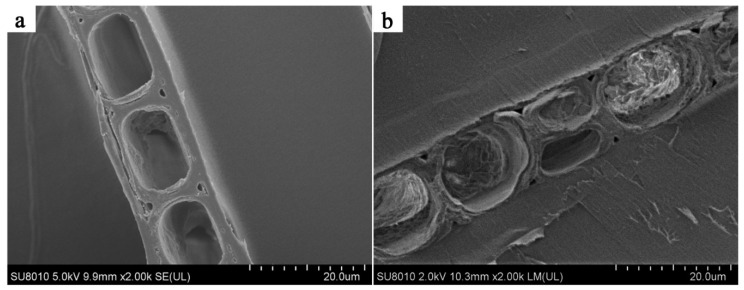
SEM photos of wood sample: (**a**) control; (**b**) impregnated.

The SEM micrographs of the Chinese fir wood samples are shown in Figure 4. The tracheids and ray cells of the wood were clean and free of sediment (Figure 4a). When impregnated with the shellac solution, some shellac was deposited in the microcapillaries, such as the wood ray cell cavity. However, there was no visible deposit in the tracheid cavity, as shown in Figure 4b.

### 3.2. Interaction Effect of Impregnation Pressure and Time

Table 2 presents the results of 16 experiments based on the RSM experiment model. Subsequently, depending on the statistics parameters, various statistical analysis approaches were utilized to select a fitting model. According to the sequential model sum of squares, the models were selected based on the highest order polynomials where the additional terms were significant and the models were not aliased. The quadratic model was suggested for all three responses of Δ*SCPG*, Δ*MOR*, and *WGP* by the software due to the sequential *p*-value (Table 3). The quadratic models of Δ*SCPG*, Δ*MOR*, and *WGP* were obtained by analysis software and given as follows:

(4) $$\Delta CSPG=-127.13+169.75A+14.45B-4.72AB-34.71A^{2}-0.30B^{2}$$

(5) $$\Delta MOR=-63.55+113.56A+2.28B-094AB-60.88A^{2}-0.05B^{2}$$

(6) $$WGP=9.63+11.86A+1.02B+0.03AB-6.30A^{2}-0.02B^{2}$$

Table 4 shows the ANOVA data of the quadratic models. The ANOVA data for the quadratic model of Δ*SCPG* revealed that it had a very low probability (*p* < 0.00001), high R-squared coefficient (R^2^ = 0.9629), adjusted R-squared coefficient (Adj-R^2^ = 0.9444), and adequate precision (19.84). The polynomial equation of Δ*MOR* was analyzed by ANOVA with a very low probability value (*p* < 0.0001), high R-squared coefficient (R^2^ = 0.9696), adjusted R-squared coefficient (Adj-R^2^ = 0.9545), and adequate precision (18.92). Additionally, the quadratic model of *WGP* had a very low probability (*p* < 0.00001), high R-squared coefficient (R^2^ = 0.9371), adjusted R-squared coefficient (Adj-R^2^ = 0.9056), and adequate precision (16.15).

By analyzing the F-value and *p*-values from Table 3, it can be found that all regression models were statistically significant (*p* < 0.0001) and the lack of fit was not significant. The Δ*SCPG* of the wood samples was profoundly (*p* < 0.0001) affected by the impregnation time (B), the interaction between the impregnation pressure and time (AB), and the quadratic term of time (B2). At the *p* < 0.005 level, impregnation pressure (A) significantly affected the Δ*SCPG*. With a *p*-value less than 0.1, the quadratic term of the pressure (B2) had less effect on the Δ*SCPG*. The impregnation time (B) and the quadratic terms of pressure (A2) and time (B2) all exhibited a very significant (*p* < 0.0001) effect on the Δ*MOR*. The impregnation pressure (A) had a modest (*p* < 0.05) influence on the Δ*MOR*, whereas the pressure and time interaction (AB) had a significant (*p* < 0.001) effect on the Δ*MOR*. At *p* < 0.0001, both the impregnation time (B) and its quadratic term (B2) had a significant effect on the *WGP*. The impregnation pressure significantly affected *WGP* at *p* < 0.005, and its quadratic term (A2) had a moderate impact on the *WGP* at *p* < 0.005. However, the interaction impact of impregnation pressure and time (AB) on the *WGP* was not significant (*p* > 0.5).

### 3.3. Effect of the Impregnation Pressure

**Figure 5:**
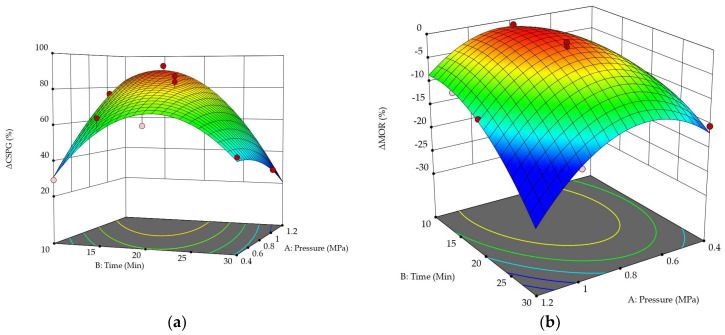
Three-dimensional surface and contour plot showing the effects of impregnation pressure and impregnation time on Δ*SCPG* and Δ*MOR*: (**a**) Δ*SCPG*; (**b**) Δ*MOR*.

**Figure 6:**
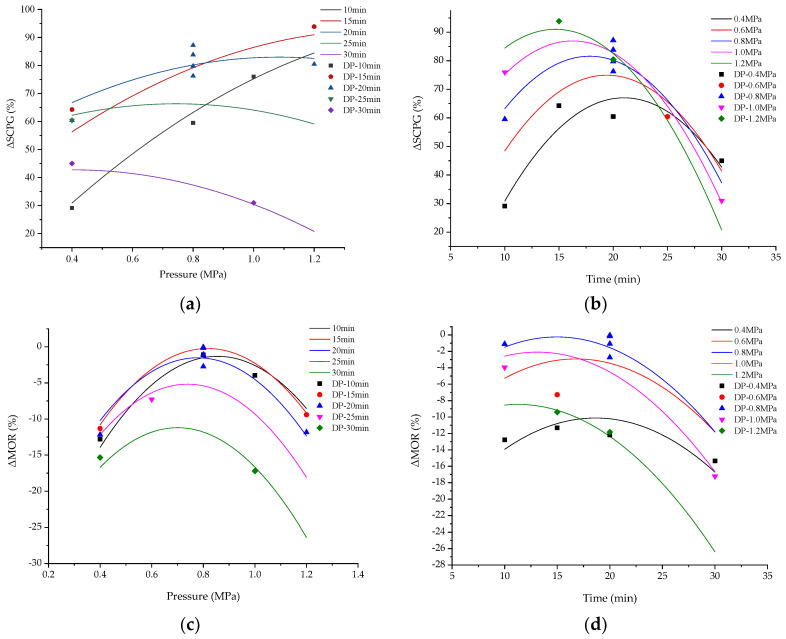
Effect of variables on Δ*SCPG* and Δ*MOR*: (**a**) effect of pressure on Δ*SCPG*; (**b**) effect of time on Δ*SCPG*; (**c**) effect of pressure on Δ*MOR*; (**d**) effect of time on Δ*MOR*. Note: The mark of “DP” in the legend indicates the design point in the RSE.

The interactions between the impregnated pressure and time on the Δ*SCPG* and Δ*MOR* of wood samples can be shown by response surface 3-dimensional (3D) plots (Figure 5). Figure 5a depicts the interaction between the impregnation pressure and time on Δ*SCPG* of the wood samples. With the impregnation time was constant, the variation of Δ*SCPG* with different impregnation pressure is shown in Figure 6a. When the impregnation time was short, the Δ*SCPG* increased significantly with an increase in the impregnation pressure, but the increase in Δ*SCPG* gradually decreased with the extension of impregnation time. When the impregnation time was 30 min, Δ*SCPG* decreased with the increase in impregnation pressure. With the impregnation pressure constant, the change of Δ*SCPG* with different impregnation times is shown in Figure 6b. It can be seen that the curves of Δ*SCPG* as a function of impregnation time at different pressures were basically parabolas, and their extreme values were approximately in the range of impregnation time of 15–20 min. With the increase in the impregnation pressure, the extreme point of the Δ*SCPG* curve moved to the left, that is, the impregnation time gradually shortened, and the Δ*SCPG* decreased faster after passing the extreme point.

Figure 5b shows the interaction between the impregnation pressure and time on Δ*MOR* of the wood samples. As the pressure increased from 0.4 MPa to 0.8 MPa, the Δ*MOR* increased from −12.17% to −0.81%, whereas it declined from −0.81% to −11.83% as the pressure increased from 0.8 MPa to 1.2 MPa. With the impregnation time constant, the variation of Δ*MOR* with different impregnation pressure is shown in Figure 6c. It was found that the Δ*MOR* increased as the impregnation pressure rose from 0.4 MPa to 0.8 MPa, whereas it reduced as the impregnation pressure increased from 0.8 MPa to 1.2 MPa, with the extreme points being around 0.8 MPa. Furthermore, when the impregnation time was extended, the extreme point shifted closer to the coordinate’s origin. This indicates that it is necessary to reduce the impregnation pressure to maintain the MOR if the impregnation time is prolonged. With the impregnation pressure constant, the variation of Δ*MOR* with different impregnation time is shown in Figure 6d. If the impregnation pressure was less than or equal to 1.0 MPa, the extreme points of the Δ*MOR* curve were between 15 min and 20 min. If the pressure was 1.2 MPa, Δ*MOR* decreased dramatically as the impregnation time increased.

**Figure 7:**
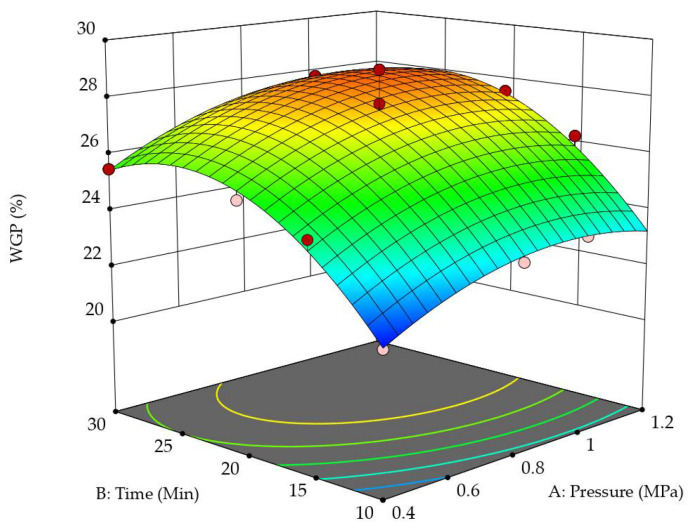
Three-dimensional surface and contour plot showing the effects of impregnation pressure and impregnation time on *WGP*.

As seen in Figure 7, WRP increased with both impregnation pressure and time. This occurs because the number of shellac molecules entering the wood interior through the tracheid lumens and pit canals increases with increasing impregnation pressure and time. However, a continuous increase in WGP does not necessarily improve the *SCPG* and *MOR* of the wood samples.

### 3.4. Optimization and Validation

Based on the fit models of Δ*CSPG* and Δ*MOR*, the impregnation pressure of [0.4, 1.2] and the impregnation time [10,30] were optimized to obtain the maximum of Δ*CSPG* and Δ*MOR*. The corresponding conditions were 0.99 MPa and 16.10 min for the impregnation pressure and the impregnation time, respectively. For verification, 12 groups of Chinese fir wood samples were impregnated with the optimized impregnation pressure and time. In order to facilitate the immersion process, the impregnation pressure was adjusted from 0.99 MPa to 1.0 MPa, and the impregnation time was adjusted from 16.10 min to 16 min. The confirmatory experiment results (Table 5) show that the Δ*SCPG* and Δ*MOR* are close to the model predicted values with low standard deviation. The measured value of Δ*SCPG* is within the 95% confidence interval. Although the mean of Δ*MOR* is outside the 95% confidence interval, the Δ*MOR* is higher than the predicted mean.

### 3.5. FTIR Spectroscopy Analysis

**Figure 8:**
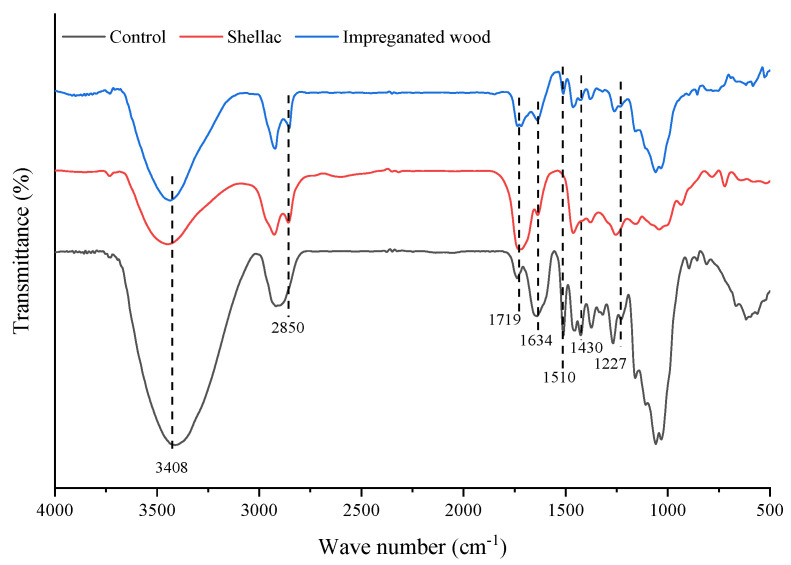
FTIR spectroscopy of the wood samples impregnated with shellac solution.

The FTIR spectra of the wood sample impregnated with shellac solution are shown in Figure 8. A comparison between the impregnated wood, pure shellac, and control wood sample reveals that the FTIR spectra of the impregnated sample have an alkane –CH_2_ stretching vibration absorption peak at 2850 cm^−1^. The -C=O stretching vibration absorption peak at 1719 cm^−1^ was enhanced. However, the 3408 cm^−1^ -OH stretching vibration peak, the 1634 cm^−1^ C=O stretching vibration absorption peak, the aromatic ring skeleton stretching vibration absorption peak at 1510 cm^−1^, the 1430 cm^−1^ -OH bending vibration peak, and the 1227 cm^−1^ aromatic ring ether bond stretching vibration absorption peak were all weakened significantly. These results mean that the penetration of shellac introduced the alkane –CH_2_, reducing the relative amounts of -OH and C=O in the wood, whereas other chemical groups did not obviously change. The results of FTIR spectrum analysis showed that there was no chemical reaction between the shellac molecules and the wood components, and the shellac molecules filled the wood cell cavity or adhered to the cell wall, which improved the compressive strength of the impregnated wood.

### 3.6. X-ray Photoelectron Spectroscopy (XPS) Analysis

**Figure 9:**
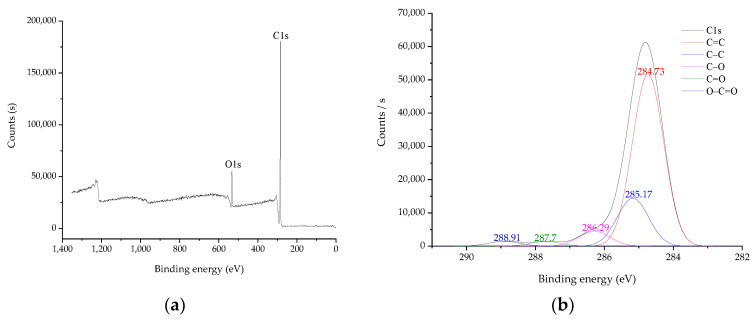

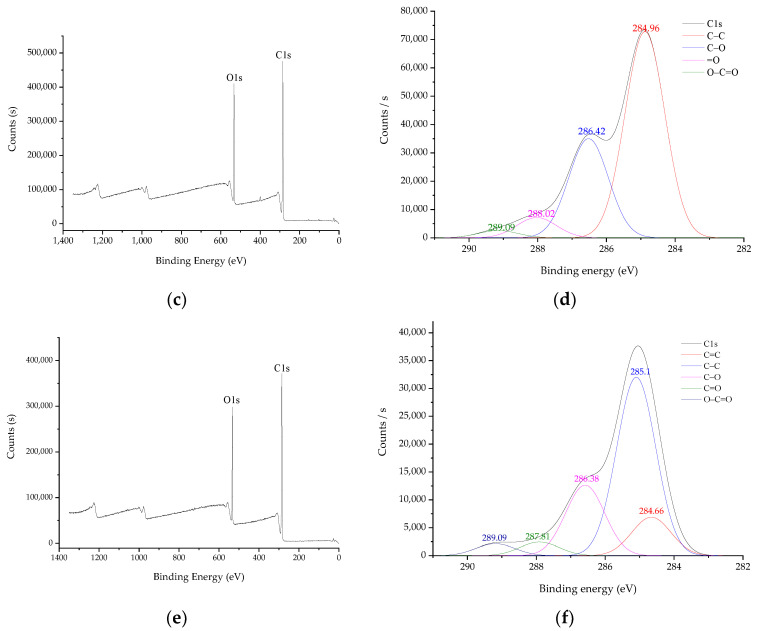
XPS spectra of shellac and Chinese fir impregnated with shellac solution under vacuum: (**a**) full spectrum of shellac; (**b**) C1s spectrum of shellac; (**c**) the full spectrum of the control wood sample; (**d**) C1s spectrum of the control wood sample; (**e**) the full spectrum of the impregnated wood sample; (**f**) C1s spectrum of the impregnated wood sample.

The full-spectrum scanning photoelectron spectra of the shellac, control wood sample, and impregnated wood sample obtained by scanning with Thermo Scientific K-Alpha photoelectron spectrometer are shown in Figure 9a, Figure 9c, and Figure 9e, respectively. There are two main elements, C and O, on the full spectrum. The results of the C1s peak fitting showed five distinctive peaks at 284.73 eV, 285.17 eV, 286.29 eV, 287.7 eV, and 288.91 eV, which are the characteristic peaks of shellac chemical structure C=C, C-C, C-O, C=O, and O-C=O [30,31], respectively, as shown in Figure 9b.

The untreated wood sample exhibited four peaks around 284.96 eV, 286.42 eV, 288.02 eV, and 289.09 eV, which are characteristic peaks of C-C, C-O, C=O, and O-C=O (Figure 9d). After impregnation with the shellac solution, the wood sample has a characteristic peak derived from C=C of shellac. The proportion of each C1s peak area of impregnated and unimpregnated wood was calculated, as shown in Table 6. Because the penetration of shellac introduced the C=C chemical structure, the proportion of C-C, C-O, and C=O peak areas of the wood samples impregnated with shellac solution relatively decreased by 21.90%, 22.93%, and 30.00%, respectively. The shellac molecules impregnated into wood samples and adhered tightly to the cell wall, effectively increasing the *SCPG* of wood samples despite having no positive effect on the *MOR*.

## 4. Conclusions

Compared to wood impregnated with different concentrations of shellac solution, wood impregnated with 15% shellac solution had a 39.01% higher *SCPG*. However, the *MOR* of the wood samples consistently decreased regardless of the concentration of the shellac solution, but the decrease in *MOR* was quite slight when impregnated with 15% shellac solution. The impregnation pressure and time were optimized by the use of response surface models. As a result of ANOVA analysis, ∆*SCPG* was significantly affected by impregnation pressure, time, and their interaction. In addition, the impregnation pressure and the interaction between impregnation time and pressure affected the ∆*MOR* of the wood samples significantly. As a result of optimization of the impregnation variables by response surface models, 1.0 MPa impregnation pressure and 16.0 min impregnation time were found to be the optimal parameters. The confirmatory experiments confirmed the response surface optimization results with low prediction errors and standard deviations, and the ∆*SCPG* was 85.78%. Shellac can be seen deposited inside the ray cell lumen and covering the inner wall of the tracheid, which could also explain the increase in the ∆*SCPG*.

## Figures and Tables

**Table 1:** Test factors and level schedule for RSM.

Factors	Symbol	Levels				
		−1	−0.5	0	0.5	1
Impregnation pressure/MPa	A	0.4	0.6	0.8	1.0	1.2
Impregnation time/min	B	10	15	20	25	30

**Table 2:** Results of the response surface experiment.

Run	A-Pressure (MPa)	B-Time (Min)	Δ*SCPG* (%)		Δ*MOR* (%)		*WGP* (%)	
			Actual	Predicted	Actual	Predicted	Actual	Predicted
1	1	30	30.96	30.43	−17.22	−16.66	27.77	27.72
2	0.8	20	87.20	80.20	−2.73	−1.54	26.56	27.54
3	1	10	75.99	75.25	−3.96	−2.58	23.55	23.57
4	0.4	10	29.14	30.89	−12.78	−13.93	21.55	21.59
5	0.4	20	60.45	66.74	−12.17	−10.21	25.30	25.61
6	0.8	20	83.83	80.20	−0.04	−1.54	28.96	27.54
7	1.2	15	93.86	90.99	−9.41	−9.17	26.23	25.87
8	1	30	30.96	30.43	−17.22	−16.66	27.77	27.72
9	0.4	30	45.01	42.76	−15.34	−16.71	25.48	25.43
10	1.2	20	80.44	82.54	−11.83	−12.36	26.98	27.46
11	0.4	15	64.27	56.30	−11.32	−10.79	24.49	24.13
12	0.6	25	60.42	65.62	−7.28	−6.36	27.15	27.29
13	0.8	20	79.76	80.20	−1.08	−1.54	27.16	27.54
14	1.2	20	80.44	82.54	−11.83	−12.36	27.53	27.46
15	0.8	20	76.25	80.20	−0.16	−1.54	27.77	27.54
16	0.8	10	59.53	63.24	−1.10	−1.50	23.19	23.42

**Table 3:** Model summary statistics.

Response	Source	Sequential *p*-Value	Lack of Fit *p*-Value	Adjusted R²	Predicted R²	Comments
Δ*SCPG*	Linear	0.0994	0.0004	0.1911	−0.1727	
	2FI	0.0380	0.0007	0.3968	−0.2417	
	Quadratic	<0.0001	0.1552	0.9444	0.9001	Suggested
	Cubic	0.2107	0.1579	0.9605	−7.3932	
	Quartic	0.1579		0.9694		Aliased
Δ*MOR*	Linear	0.1823	0.0002	0.1120	−0.1089	
	2FI	0.2601	0.0002	0.1383	−0.4856	
	Quadratic	<0.0001	0.1475	0.9545	0.8173	Suggested
	Cubic	0.0618	0.9693	0.9796	0.9816	
	Quartic	0.9693		0.9756		Aliased
*WGP*	Linear	0.0010	0.0990	0.6024	0.4821	
	2FI	0.9582	0.0790	0.5694	0.1053	
	Quadratic	0.0002	0.9655	0.9056	0.8914	Suggested
	Cubic	0.9137	0.8238	0.8633	0.0108	
	Quartic	0.8238		0.8378		Aliased

**Table 4:** Analysis of variance (ANOVA) for the quadratic models of Δ*MOR*, Δ*SCPG*, and *WGP*.

Response	Source	Sum of Squares	df	Mean Square	F-Value	*p*-Value	Comments
Δ*SCPG*	Model	6448.94	5	1289.79	51.94	<0.0001	significant
	A-Pressure	474.22	1	474.22	19.10	0.0014	significant
	B-Time	1102.95	1	1102.95	44.41	<0.0001	significant
	AB	1132.99	1	1132.99	45.62	<0.0001	significant
	A²	97.19	1	97.19	3.91	0.0761	
	B²	2917.24	1	2917.24	117.47	<0.0001	significant
	Residual	248.33	10	24.83			
	Lack of Fit	180.09	5	36.02	2.64	0.1552	not significant
	Pure Error	68.24	5	13.65			
Δ*MOR*	Model	550.68	5	110.14	63.87	<0.0001	significant
	A-Pressure	8.75	1	8.75	5.07	0.0480	
	B-Time	174.54	1	174.54	101.21	<0.0001	significant
	AB	45.05	1	45.05	26.12	0.0005	significant
	A²	298.97	1	298.97	173.37	<0.0001	significant
	B²	85.01	1	85.01	49.30	<0.0001	significant
	Residual	17.24	10	1.72			
	Lack of Fit	12.62	5	2.52	2.73	0.1475	not significant
	Pure Error	4.63	5	0.9253			
*WGP*	Model	57.26	5	11.45	29.78	<0.0001	significant
	A-Pressure	6.47	1	6.47	16.82	0.0021	significant
	B-Time	26.89	1	26.89	69.91	<0.0001	significant
	AB	0.0347	1	0.0347	0.0902	0.7701	
	A²	3.20	1	3.20	8.32	0.0163	
	B²	14.42	1	14.42	37.50	0.0001	significant
	Residual	3.85	10	0.3846			
	Lack of Fit	0.5415	5	0.1083	0.1639	0.9655	not significant
	Pure Error	3.30	5	0.6609			

**Table 5:** Statistical parameters of confirmatory test results.

Response	Predicted Mean	Std. Dev.	SE Pred	95% PI Low	Data Mean	95% PI High
Δ*CSPG*	86.7814	4.9833	2.8447	80.4429	85.7800	93.1198
Δ*MOR*	−2.3916	1.3132	0.7496	−4.0619	−0.1850	−0.7213
*WGP*	26.6169	0.6201	0.3540	25.8281	25.2300	27.4057

**Table 6:** The ratio of chemical structure and C1s peak of the wood impregnated with shellac solution.

Sample	Index	Chemical Structure				
		C=C	C-C	C-O	C=O	O-C=O
Impregnated wood	Binding energy/eV	284.66	285.10	286.39	287.76	289.01
	Ratio of the peak area/%	24.1	48.38	22.96	4.27	3.79
Control	Binding energy/eV	-	284.96	286.42	288.02	289.09
	Ratio of the peak area/%	-	61.95	29.79	6.10	2.16

## Data Availability

Not applicable.
